# Complete Mitogenome Assembly and Comparative Analysis of *Vaccinium bracteatum* (Ericaceae), a Rich Source of Health-Promoting Molecules

**DOI:** 10.3390/ijms252212027

**Published:** 2024-11-08

**Authors:** Peng Zhou, Fei Li, Qiang Zhang, Min Zhang

**Affiliations:** 1 Jiangsu Academy of Forestry, 109 Danyang Road, Dongshanqiao, Nanjing 211153, China; zpjsslky@163.com (P.Z.); 13886176450@163.com (F.L.); 2 Co-Innovation Center for Sustainable Forestry in Southern China, Key Laboratory of State Forestry and Grassland Administration on Subtropical Forest Biodiversity Conservation, College of Life Sciences, Nanjing Forestry University, Nanjing 210037, China

**Keywords:** *vaccinium bracteatum*, mitogenome, comparative analysis, phylogenetic analysis, repeat sequence, molecular breeding

## Abstract

*Vaccinium bracteatum* is a valuable plant used both as food and medicine in China, but low production limits the development of its industry. As such, it is important to develop genetic resources for the high-value species for preservation of wild populations and utilization. The complete chloroplast and nuclear genomes have already been available; however, its mitogenome has not yet been characterized. Here, the *V. bracteatum* mitogenome was assembled using HiFi reads, and a comparative analysis was conducted. The mitogenome was a circular sequence of 708,384 bp with a GC content of 45.28%, in which 67 genes were annotated, including 36 protein-coding genes, 26 tRNA genes, 3 rRNA genes, and 2 pseudogenes. Overall, 370 dispersed repeats, 161 simple repeats, and 42 tandem repeats were identified, and 360 RNA editing sites were predicted. There was extensive DNA migration among the three genomes. In addition, most of the protein-coding genes underwent purifying selection throughout evolution, and the nucleotide diversity was highly variable. In addition, comparative analysis indicated that the sizes, structures, and gene contents of the mitogenomes differed significantly, but the GC contents and functional genes were relatively conserved among the Ericales species. Mitogenome-based phylogenetic analysis indicated the precise. evolutionary and taxonomic status of *V. bracteatum*. The complete mitogenome represents the last link of the reference genome of *V. bracteatum* and lays the foundation for effective utilization and molecular breeding of this plant.

## 1. Introduction

The genus *Vaccinium* Linn. of the Ericaceae family encompasses a vast array of deciduous or evergreen species, with approximately 450 species found in cool areas of the northern and southern hemispheres [1,2,3]. Several species are identified as widely recognized berry crops of high commercial importance, such as the blueberry (*V. corymbosum, V. ashei*), lingonberry (*V. vitis-idaea*), and bilberry (*V. myrtillus*) [4,5,6]. Moreover, as global demand for healthy foods continues to rise, researchers are increasingly turning their attention to wild relatives, which contain increased levels of bioactive compounds [5].

*V. bracteatum* Thunb. (named wu fan shu in China) is a rare small arbor or evergreen shrub with dark purple fruits, growing in the hilly regions of East Asia, especially in the east and south of China [7,8,9,10]. This species has been widely used in various traditional medicines and food products, it contains a series of unique bioactive compounds, and its berries are used to produce juice, jam, and vinegar in the food industry [11,12]. Moreover, previous studies have established that extracts from *V. bracteatum* leaves (VBLs) had a hypoglycemic effect, tyrosinase inhibition, antioxidant activity, and anti-inflammatory activity [13]. In China, for over a thousand years, VBLs have been utilized both as natural medicines and functional foods to nourish the body and promote longevity [4], and also as food preservatives [14]. Until now, residents of the Yangtze River basin, e.g., Jiangsu Province and Anhui Province, have also maintained the tradition of eating ‘wu fan’ every spring, which is a traditional food with a hypoglycemic effect made from VBL juice and rice [10,12,15,16], and has evolved into a popular local tourism food [17]. The consumption regions are now extended to most areas of the eastern and southern regions of China. 

As an important medicinal and economic plant, *V. bracteatum* has enormous economic and development potential, while the cultivation of this species as an economic plant only emerged in the past decade and efforts to improve cultivars are still in the early stages [2,17]. In recent years, *V. bracteatum* has gained more and more attention; however, low production limits the development of the wu fan industry. Due to the absence of enhanced varieties, the cultivation and industrialization of *V. bracteatum* are restricted, which means that there is an immediate demand for high-quality and high-yield varieties in production. Reasonably developing germplasm resources is the key to realizing the goal of modern breeding. Hybridization technology related to cytoplasmic male sterility (CMS) associated with various chimeric open reading frames (ORFs) in the plant mitogenome can produce modern cultivars with significant heterosis and presents a viable approach to preserving higher crop yields [18]. Unfortunately, no mitogenome of *V. bracteatum* has been reported thus far, which severely restricts follow-up research. 

Furthermore, in recent years, populations of this species have been under pressure from increasing demand and anthropogenic activities, causing widespread habitat loss. Molecular biology and genetics studies can lay the groundwork for the conservation and utilization of this species [19]. As such, it is crucial to enhance genomic resources to aid in tracking illegal logging and clarify the phylogeny of this species, gain a deeper understanding of its population status, and, subsequently, develop conservation-genomics-based strategies for the preservation of threatened wild populations.

Higher plants possess three genomes: one located in the nucleus and two within organelles. The nuclear (nu) genome contains the vast majority of genetic information, while the organelles, especially the chloroplasts (cp) and mitochondria (mt), also harbor independent genetic material [20,21]. These multi-copy organellar genomes serve as valuable resources for phylogenetic studies, population genetics, and genetic improvement [5,22,23]. Recently, the complete cp and nu genomes of *V. bracteatum* have been successfully assembled and annotated (GenBank Accession number: LC521967.1) [24]; however, the complete genomic profile remains unresolved until the mitogenome is sequenced and assembled. In contrast to chloroplast genomes, the mitogenome exhibits considerable size and structure variation [20], which primarily arise from an extensive quantity of foreign DNA and repetitive sequences [22]. Other characteristics of plant mitogenomes include the available molecular markers [25], haploid and matrilineal inheritance [26], low mutation rate [26,27], highly variable non-coding regions [18], and the homology of coding genes [28]. Thus, mitogenomes can provide solutions for elucidating previously intractable phylogenies and interspecies discrimination [29]. 

In addition, the mitogenome can be utilized to produce hybrids with substantial heterogeneity, because CMS is triggered by specific mitochondrial mutations [30,31]. Therefore, mitogenomes are valuable genetic resources for plant systematics and essential cellular process studies. Decoding complete plant mitogenomes is highly significant for understanding genetic variation, evolutionary mechanisms, species identification, and the molecular breeding of species [31,32]. However, in comparison to chloroplast genomes, the sequencing and assembly of mitogenomes faces a challenge because of the structural complexity of plant mitogenomes, which is marked by the deposition of repetitive sequences, integration of chloroplast DNA, and extensive rearrangements [33,34]. To date, mitogenomes of only two species of *Vaccinium*, *V. macrocarpon* (Genbank: NC_023338.1) and *V. microcarpum* (Genbank: MK715445.1), have been submitted to the NCBI. In comparison to the numerous *Vaccinium* species, the number of sequenced mitogenomes within this genus remains limited, which may significantly impede a comprehensive understanding of mitogenome evolution within this extensive family. Fortunately, the sequencing and assembly of mitogenomes has become more feasible and cost-effective in recent years [28,31,35,36].

In this study, we conducted the following: (1) using the whole genome sequencing data from the PacBio HiFi platform, we assembled and annotated the *V. bracteatum* mitogenome; (2) characterized the genomic features, including the gene content, codon usage, and RNA editing sites; (3) compared the mitogenome with other published Ericales species and performed a phylogenetic analysis; and (4) searched homologous fragments to assess sequence transfer among nuclear and organellar genomes. These findings will enhance our understanding of the structure and function of the *V. bracteatum* mitogenome and offer insights for research in conservation biology, population genetics, evolutionary history, and the breeding of new varieties of this important edible and medicinal species.

## 2. Results

### 2.1. Mitogenome Annotation and Structure Characterization

**Figure 1:**
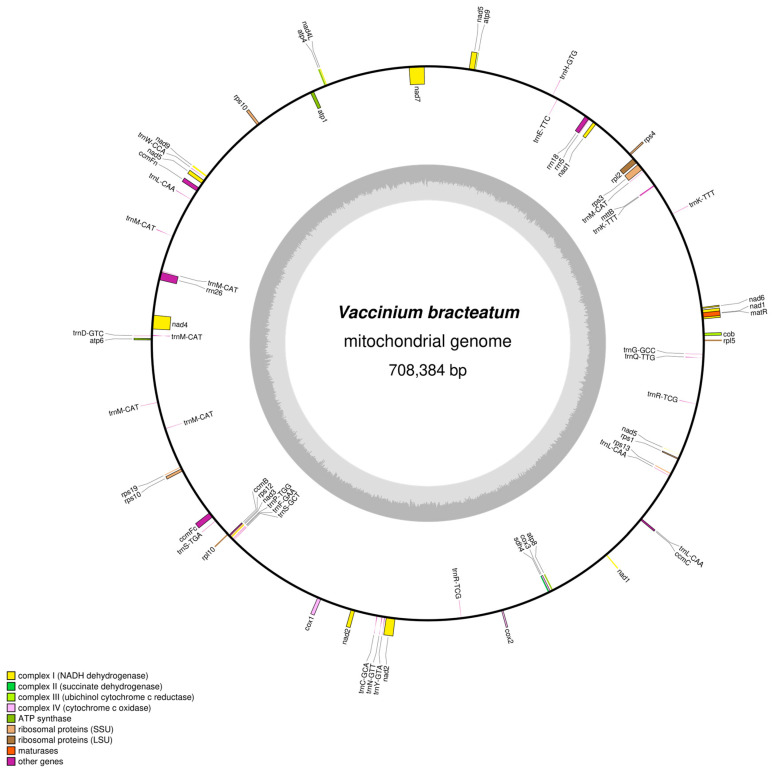
Circular map of the *V. bracteatum* mitogenome. Genes shown on the outside and inside of the circle are transcribed clockwise and counterclockwise, respectively. The dark gray region in the inner circle depicts the GC content.

Using the sequences obtained from long PacBio HiFi data, we successfully assembled the entire mitogenome of *V. bracteatum*, which formed a circular sequence spanning 708,384 bp. The annotated results showed a total of 67 genes, containing 36 protein-coding genes (PCGs), 26 tRNA genes, 3 rRNA genes, and 2 pseudogenes (*rpl16* and *rps14*), in the *V. bracteatum* mitogenome (Figure 1; Table 1). Additionally, 4078 ORFs were identified. The nucleotide compositions were A (27.31%), T (27.42%), C (22.67%), and G (22.61%) (Supplementary File S1: Table S1). The complete mitogenome exhibited a GC percentage of 45.28%, with 42.46% in PCGs, 50.36% in rRNAs, and 51.50% in tRNAs. Remarkably, the GC content in the PCGs was observed to be lower than that of other regions. There was a positive GC skew observed in both the mitogenome and the CDS regions.

The overall length of all of the PCGs was 30,906 bp, and the mitogenome encoded thirty-five different proteins (*rps10* has two copies) that were classified into 10 groups (Table 1), including five ATP synthase genes, four cytochrome C biogenesis genes, a ubiquinol cytochrome c reductase gene, three cytochrome C oxidase genes, a maturase gene, a transport membrane protein gene, nine NADH dehydrogenase genes, three ribosomal protein (LSU) genes, seven ribosomal protein (SSU) genes, and a succinate dehydrogenase gene.

Three rRNA genes, including *rrn18* (1935 bp), *rrn26* (3343 bp), and *rrn5* (120 bp), were annotated. Moreover, seventeen tRNA genes (with *trnK-TTT* and *trnR-TCG* each presented as two copies, *trnL-CAA* presented as three copies, and *trnM-CAT* presented as six copies) were annotated in the *V. bracteatum* mitogenome. The length of tRNAs varied from 70 to 88 bp, contributing to an overall length of 1966 bp.

Fifty-three genes have no introns, while the other fourteen annotated genes contained twenty-seven introns (Table 1): *nad1*, *nad2*, *nad5*, and *nad7* had four introns, followed by *nad4* (2); *ccmFc*, *rpl2*, *rps1*, *rps10*, *rps3*, and *trnK-TTT*; and *trnR-TCG* (1). 

### 2.2. Repeat Sequence Analysis

**Figure 2:**
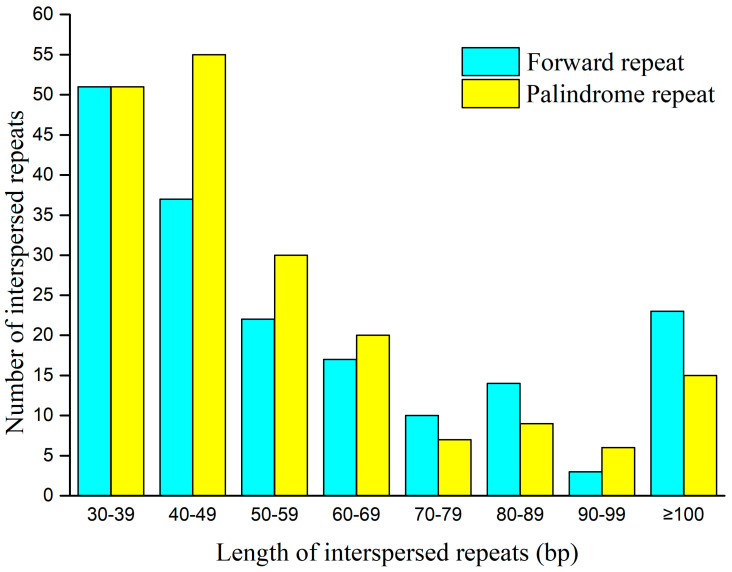
The length distribution of interspersed repeats in the *V. bracteatum* mitogenome.

Supplementary File S2: Figure S2 displays three varieties of repetitive sequences present in *V. bracteatum*. Dispersed repeats are repetitive sequences spread unevenly across the genome [37]. A total of 370 dispersed repeats were identified (Supplementary File S1: Table S2), accounting for 8.98% (63,598 bp). Notably, 177 and 193 were forward repeats and palindromic repeats, respectively. The longest forward repeat had a length of 29,795 bp, whereas the longest palindromic repeat was 534 bp. The length of the forward repeats mainly ranged from 30–39 bp, whereas the length of the palindromic repeats was most prevalent between 40–49 bp (Figure 2).

SSRs are simple repetitive sequences of 1–6 bp repeated multiple times and distributed throughout the genome of eukaryotes [33,37]. As shown in Supplementary File S1: Table S3, 161 SSRs were detected in the *V. bracteatum* mitogenome, and the detected SSR sites included monomers, dimers, trimers, tetramers, and pentamers. Tetramer repeats were the most prevalent type, constituting 39.13%, followed by dimer and monomer repeats, which represented 24.84% and 19.88%, respectively; the numbers of pentamer and hexanucleotide repeats were the lowest. A/T base repeats accounted for 96.88% of monomer SSRs, and AG/CT bases repeats constituted 72.50% of dimer SSRs.

Tandem repeats are DNA fragments with multiple copies of core repeating units of ≥ 7 bases adjacent to one another [18]. As shown in Supplementary File S1: Table S4, 42 tandem repeats, with a range of 9–71 bp in length and exhibiting a matching degree > 72%, were identified in the genome.

### 2.3. RNA Editing Sites Prediction

**Figure 3:**
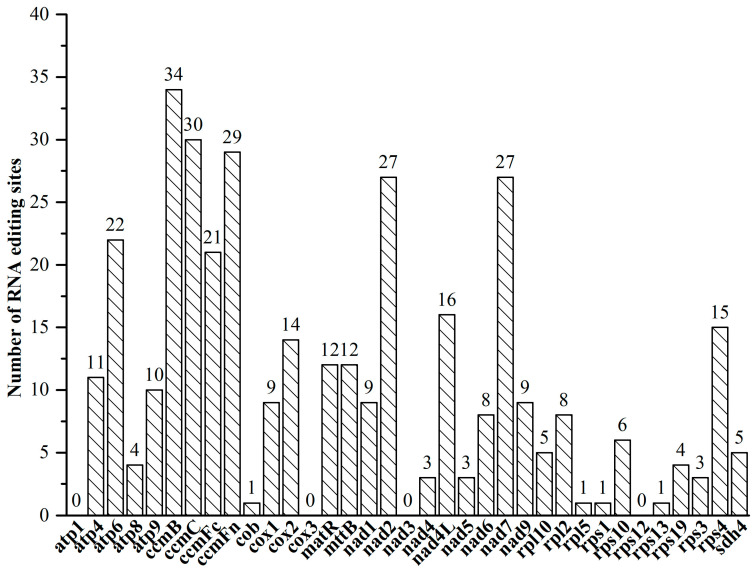
RNA editing sites in the PCGs of the *V. bracteatum* mitogenome.

In this study, 360 RNA editing sites were predicted within 36 PCGs of the *V. bracteatum* mitogenome (Supplementary File S1: Table S5). As shown in Figure 3, the highest quantity of RNA editing sites was predicted in *ccmB* (34), followed by *ccmC* (30) and *ccmFn* (29). Only one editing site was predicted in *cob*, *rpl5*, *rps1*, and *rps13*. No potential RNA editing sites were predicted in genes *atp1*, *cox3*, *nad3*, and *rps12*, and in two pseudogenes (*rpl16* and *rps14*). Due to RNA editing, the hydrophobicity of 40.28% of the amino acids was predicted to remain stable, 7.22% to change from hydrophobic to hydrophilic, and 51.11% to change from hydrophilic to hydrophobic.

A total of 29 types of codon transfer types were predicted, associated with 14 amino acid transfer categories (Supplementary File S1: Table S5). Among them, TCA => TTA was the most prevalent, with 58 sites identified. The results also indicated that the highest conversion tendency was to leucine, with 43.89% (158 sites) of the amino acids converted. All RNA-editing sites were of the C-T editing type, and the predicted RNA editing events all occurred at the first (121, 33.61%) or second (231, 64.17%) positions of the codons, while no editing was observed at the third position of the triplet codons. In specific editing cases, both the first and second bases of the triplet codon were edited, leading to the conversion of CCT to TTT. In addition, 1.39% of the amino acids in *atp6*, *atp9*, *ccmFc*, *rps10,* and *nsdh4* were converted to termination codons (TAA, TGA).

### 2.4. Codon Usage Analysis

The codon compositions of the *V. bracteatum* mitogenome were analyzed (Supplementary File S1: Table S6). A total of 10,181 codons were identified across all coding genes, with GC contents all being below 50%, suggesting a codon bias in the *V. bracteatum* mitogenome. Additionally, the effective codon number (Nc) was calculated to be 53.19, indicating a weak preference for codons [33]. 

To examine codon usage preference, codon usage analysis was performed (Supplementary File S1: Table S7). In the PCGs, leucine (Leu) emerged as the dominant amino acid (11.01%), followed by isoleucine (Ile) (7.86%) and glycine (Gly) (6.96%), whereas tryptophan (Trp) and cysteine (Cys) only occurred 156 and 152 times (1.53% and 1.49%, respectively). The codon UUU, encoding for phenylalanine (Phe), was the most frequently used codon (3.94%), and the UAG termination codon had the lowest frequency.

**Figure 4:**
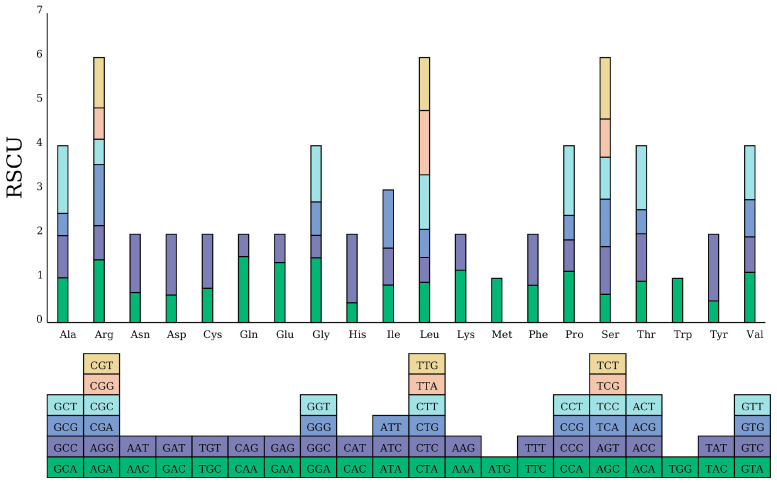
Codon usage bias analysis in the *V. bracteatum* mitogenome. The block below represents all of the codons that encode each amino acid.

The range of RSCU values in the mitogenome ranged from 0.34 (UAG in the termination codon) to 1.71 (UAA in the termination codon) (Supplementary File S1: Table S7). Except for the start codon (ATG) and tryptophan (Trp, TGG) [38], multiple codons were responsible for encoding other amino acids (Figure 4). Specifically, proline (Pro) was predominantly encoded by CCU, showing an RSCU of 1.57. The stop codon was mainly encoded by UAA, with a maximum RSCU of 1.71. There were 30 codons with RSCUs> 1, most of which end with an A/T base. In addition, with respect to the 36 PCGs, all of the PCGs began with the typical start codon ATG, and the usage rates of the stop codons TAG, TGA, and TAA were 11.11%, 33.33%, and 55.56%, respectively (Supplementary File S1: Table S8).

### 2.5. Genome Alignment and Migration Sequence

**Figure 5:**
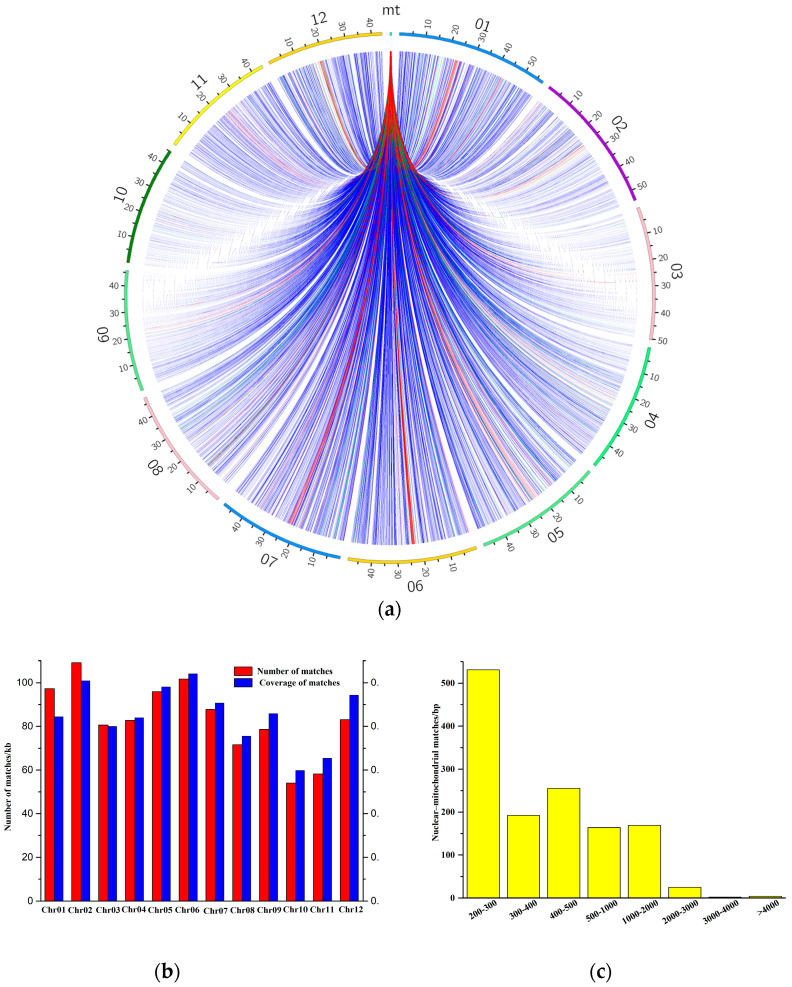
Characteristics of nuclear/mitochondrial sequences in *V. bracteatum*. (**a**) Homologous fragments between mitogenome and nuclear genome. (**b**) Distributions of nuclear/mitochondrial matches. (**c**) Distributions of lengths of nuclear/mitochondrial matches.

To investigate intergenomic fragment transfer events in *V. bracteatum*, the cp and nu genomes were searched by using its mitogenome sequences as queries [39,40]. A total of 4512 hits covering 1011.43 kb of sequences of the nuclear genome transferred into the mitogenome were obtained (Figure 5a). As shown in Figure 5b, each *V. bracteatum* chromosome exhibited hits, though the total lengths and the percent coverage of these hits differed. Chromosome 2 exhibited the longest total length of hits (109.14 kb), and the greatest percent coverage (0.21%) existed on chromosome 6. In addition, the most abundant fragment lengths were found within the range of 200–300 bp (Figure 5c). A total of 362.83 kb of sequences (51.22% of the *V. bracteatum* mitogenome) was shared between the nuclear and mitochondrial genomes (Supplementary File S1: Table S9). Numerous homologous segments within the nuclear genome contained several complete mitochondrial genes, including one PCG (*ccmFn*) and nine tRNA genes (*trnS-GCT*, *trnS-TGA*, *trnM-CAT*, *trnL-CAA*, *trnF-GAA*, *trnG-GCC*, *trnW-CCA*, *trnK-TTT*, and *trnC-GCA*).

**Figure 6:**
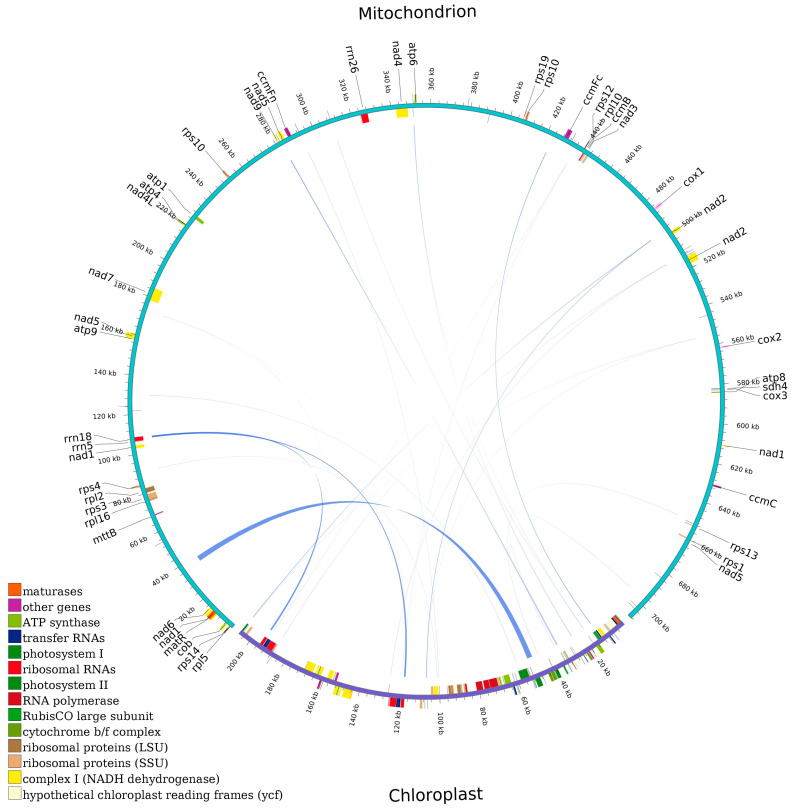
A schematic representation of the homologous fragments between the mt and cp genomes in *V. bracteatum*. The purple arcs of the circle represent the cp genome and the blue arcs represent the mitogenome. The lines between the arcs correspond to the genomic fragments that are homologous.

Homologous fragments between the mt and cp genomes were searched and analyzed (Figure 6). The *V. bracteatum* mitogenome (708,384 bp) was about 3.43 times the length of the cp genome (206,405 bp). The results showed that a total of 24 homologous fragments ranged from 35 to 2826 bp with a total length of 5500 bp, constituting 0.78% of the mitogenome, were discovered in *V. bracteatum* (Supplementary File S1: Table S10). Two intact chloroplast PCGs (*psbD* and *psbC*), four tRNA genes (*trnW-CCA*, *trnD-GUC*, *trnM-CAU,* and *trnN-GUU*), and many incomplete genes and intergenic spacer areas were matched to the mitogenome. The mitochondria, chloroplasts, and nuclei shared two homologous genes, which were *trnW-CCA* and *trnM-CAT*.

### 2.6. Phylogenetic Analysis

**Figure 7:**
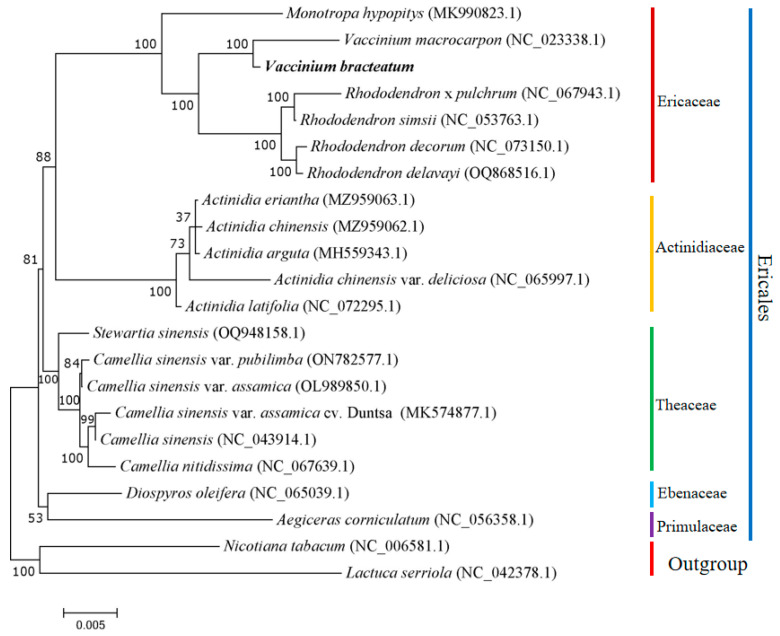
Maximum likelihood tree based on the mitogenomes of 22 Ericales species. *Nicotiana tabacum* and *Lactuca serriola* were used as outgroups. The number on each node is bootstrap support value. The evolutionary branch length represents the degree of branch variation. The number after the species name is the GenBank accession number. Colors indicate the groups to which the species belong.

Based on the mitogenomes of *V. bracteatum* and 21 other plants, a phylogenetic tree was obtained (Figure 7). As outgroups, *N. tabacum* and *L. serriola* were distinct from the Ericales species. The species from five families, Ebenaceae, Primulaceae, Theaceae, Actinidiaceae, and Ericaceae, were well clustered into five separate branches, aligning with the findings of the APG IV taxonomic framework, a modern classification system of flowering plants strongly based on molecular phylogenetics [41]. Additionally, the phylogenetic tree provided strong evidence (bootstrap support = 100%) for the strong phylogenetic association between the target tree species *V. bracteatum* and *V. macrocarpon*, which both belong to the genus *Vaccinium*, and established a sister cluster with the genus *Rhododendron* in the Ericaceae family, supported by a bootstrap support value of 100% (Figure 7). Overall, the clustering observed in the phylogenetic tree aligned with the family-level relationships of these species, suggesting that the mitogenome clustering results were reliable. Furthermore, several species were selected for further comparative analysis based on the phylogenetic tree.

### 2.7. Substitution Rates of PCGs

**Figure 8:**
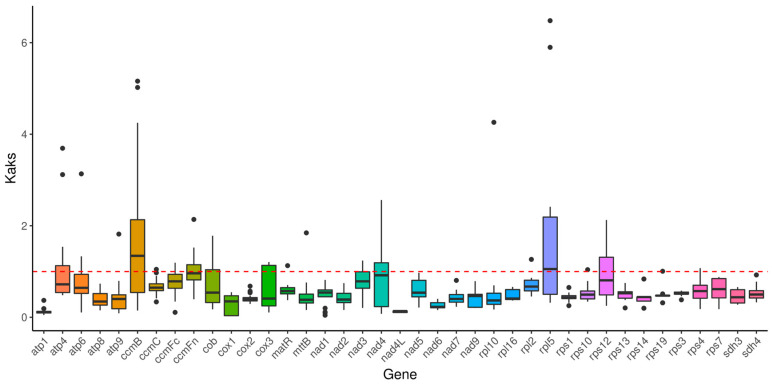
Boxplots of the Ka/Ks values of the shared PCGs among seven Ericales species.

The PCGs from the *V. bracteatum* mitogenome were compared to those of the mitogenomes of six other Ericales species for calculating Ka/Ks (Supplementary File S1: Table S11). As shown in Figure 8, Ka/Ks varied from 0.029 for *cox1* to 6.482 for *rpl5*. The gene *ccmB* showed the highest average Ka/Ks ratio (1.745). However, the majority of the genes demonstrated significantly low Ka/Ks values (< 1), implying purifying selection during evolution [42]. The gene *nad4L* exhibited the lowest Ka/Ks (0.124), indicating significant conservation throughout the evolution of Ericales plants [42].

### 2.8. Nucleotide Diversity

**Figure 9:**
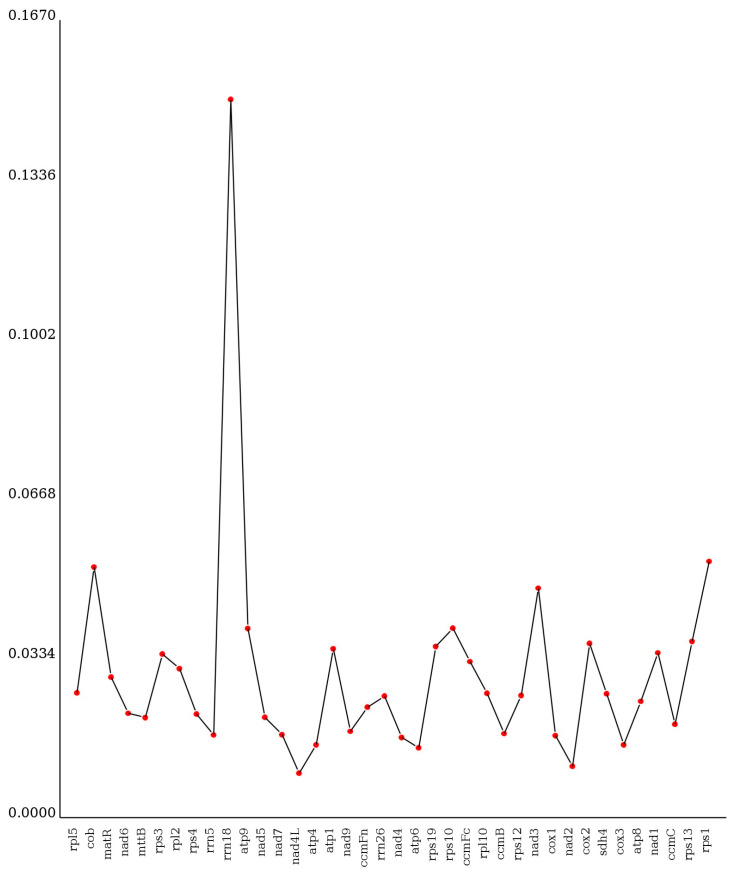
Nucleotide diversity (Pi) among Ericales mitogenomes.

The Pi values of 35 PCGs and 3 rRNA genes ranged from 0.009 to 0.150 (Figure 9; Supplementary File S1: Table S12). Among them, *rps1* (0.054) and *cob* (0.052) were highly variable. Conversely, the most conserved PCG was *nad4L* (0.009). Moreover, the Pi values of three rRNA genes varied greatly, with values of 0.017 for *rrn5*, 0.150 for *rrn18,* and 0.025 for *rrn26*. In general, the nucleotide diversity of the PCGs exhibited considerable variability across the seven Ericales mitogenomes.

### 2.9. Variation in Genome Composition

In this study, the *V. bracteatum* genome serves as a valuable resource for understanding the fundamental genomic features and gene arrangement within the Ericaceae family. To further explore the evolutionary characteristics of the mitogenome of *V. bracteatum*, we compared it to six other representative Ericales species. The sizes of these seven mitogenomes varied ranging from 425,282 bp (*Aegiceras corniculatum*) to 825,163 bp (*Actinidia latifolia*). Conversely, they had a comparable AT and GC content, ranging from 53.84 to 55.18% and 44.82 to 46.16%, respectively (Table 2). *A. corniculatum* displayed the lowest GC% and the highest AT% across all species (Table 2). *Camellia sinensis* and *A. latifolia* demonstrated negative skew values for GC and AT. The *Diospyros oleifera* mitogenome had negative and positive skew values for GC and AT, respectively. However, *V. bracteatum* has positive and negative skew values for GC and AT. Various species (*A. corniculatum*, *R. simsii,* and *V. macrocarpon*) showed positive skew values for GC and AT in the mitogenome, implying that these genomes possessed greater A bases and G bases.

With regard to the genes composing the mitogenomes, these seven species were identified with the three typical ribosomal units (*rrn5*, *rrn18,* and *rrn26*). However, among these species, obvious variation in PCGs was identified. The seven Ericales mitogenomes identified different numbers and compositions of PCGs. Within the seven species spanning five lineages of Ericales, *R. simsii* displayed the most PCGs (41), whereas *C. sinensis* had the fewest PCGs (32) (Table 2). 

**Figure 10:**
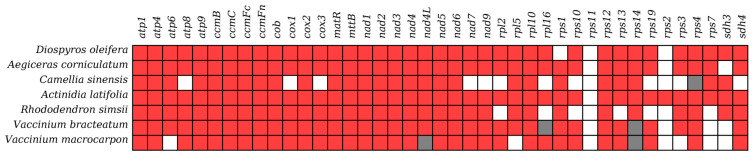
The composition of PCGs in the 7 Ericales mitogenomes.

The mitogenome of the common ancestor of angiosperms contains 41 PCGs [43]. Most of these seven species possessed a full complement of 24 PCGs classified as core genes [30]. Nevertheless, several core genes were either absent (represented by white squares in Figure 10) or presented as incomplete sequences (pseudogenes; represented by grey squares in Figure 10). *V. macrocarpon* lacked the genes *atp6* and *nad4L*, while the genes *atp8*, *cox1*, *cox3*, *nad7,* and *nad9* were not found in *C. sinensis*. Conversely, the other 17 genes called variable PCGs were highly variable across the seven studied Ericales species. Specifically, we observed the absence and pseudogenization of *rps* and *sdh*. *C. sinensis* lacked eight genes belonging to these two categories, and several Ericales species commonly lacked *rps2* and *rpl11*, with only *A. latifolia* containing *rps2*. Additionally, for seven species, we detected a total of five pseudogenes in three species (*C. sinensis*, *V. bracteatum,* and *V. macrocarpon*). The species *V. bracteatum* and *V. macrocarpon* both had two pseudogenes, while *C. sinensis* had only one (*rps4*). *V. bracteatum* and *V. macrocarpon* exhibited 31 shared PCGs.

### 2.10. Comparison of the Genome Structure

**Figure 11:**
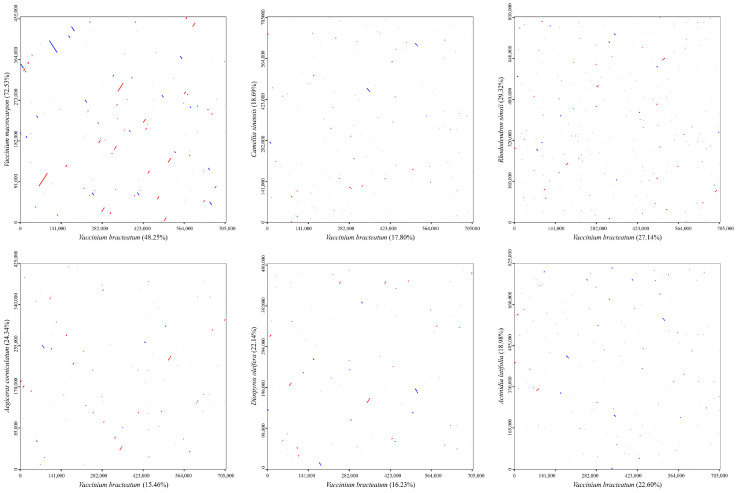
Dot-plot graphs indicating syntenic sequences between mitogenomes in Ericales species.

**Figure 12:**
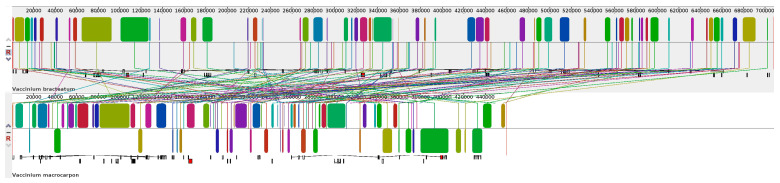
Collinearity analysis of the mitogenomes between *V. bracteatum* and *V. macrocarpon*. The same color blocks represented homologous regions.

To further evaluate the structural variations, the mitogenome of *V. bracteatum* was compared to those of six Ericales species, including one Ebenaceae, one Primulaceae, one Theaceae, one Actinidiaceae, and two Ericaceae, and the *V. bracteatum* mitogenome was used as a reference. According to the dot-plot (Figure 11), the longest syntenic sequences showed the most significant similarity between *V. bracteatum* and *V. macrocarpon*, revealing a close genetic affinity between the two species. Despite high sequence similarity, collinearity analysis (Figure 12) showed that the arrangement of homologous co-linear blocks differed between the two mitogenomes. And the arrangement of PCGs was not consistent between the two *Vaccinium* mitogenomes, with many differences (Supplementary File S2: Figure S2). These significant rearrangement events suggested that the mitogenomes exhibited extreme structural non-conservation. 

## 3. Discussion

### 3.1. Characterization of the V. bracteatum Mitogenome

The mitogenome is crucial for plant productivity and development and serves as an important source of genetic material for evolutionary research and population genetics. Furthermore, mitogenome recombination has been widely used for the study of CMS in plants [18,29,31]. Due to the intricate structure and accumulation of repetitive sequences, it is difficult to assemble the plant mitogenome poses with traditional sequencing techniques [18,44]. The development of third-generation sequencing makes it possible to quickly assemble the plant organelle genomes by employing the genomes of related species as a reference [19,45]. In this project, the PacBio HiFi reads were employed to successfully assemble the first complete mitogenome of *V. bracteatum*. Due to the high recombination frequency, plant mitogenomes exhibit multiple configurations, including single circular, multiple circular, branched, linear, and complex forms, in mitochondria [19,33]. The *V. bracteatum* mitogenome assembled was a typical single circular molecule, with a size of 708,384 bp and a GC content of 45.28%. The overall length of the *V. bracteatum* mitogenome was greater than that of *V. macrocarpon* and *V. microcarpum*, reported species from the genus *Vaccinium*, but the GC content was similar. Our result is consistent with earlier studies showing significant size variation in mitogenomes among species, even within the same family of plants [46]. Similar to other mitogenomes [39], the majority of the sequences in the *V. microcarpum* mitogenome were non-coding, with PCGs comprising only 4.36%. 

Although there are obvious changes in the size and structure of plant mitogenomes, functional genes show a degree of conservation [43,47]. Approximately 35 of the 41 PCGs found in the common ancestors of angiosperms were detected in the *V. bracteatum* mitogenome. The genes *rps2*, *rps7*, *rps11*, and *sdh3* were lost, which might have been functionally transferred to the nuclear genome [48], and the genes *rps16* and *rps14* were present as pseudogenes. Moreover, the three species shared an identical number of rRNA genes, but they differed in the quantity of tRNA genes. Compared with reported species from Ericales, the length of the *V. bracteatum* mitogenome is intermediate. In addition, there is no relationship between mitogenome length and the number of genes, as size variations are primarily due to differences in non-coding regions [9,39,49] such as repeated sequences which were commonly dispersed throughout the mitogenome of *V. bracteatum*.

Codon usage bias is a very important factor reflecting the genetic evolution of mitogenomes [38]. The rate of codon usage varies significantly among different species [22,50], and the differences in codon usage preferences may be determined by several factors, such as natural selection, mutation, and phylogenetic relationship [51]. In the *V. bracteatum* mitogenome, all PCGs typically begin with ATG start codons, which is similar to other higher angiosperms [38], and there were 30 codons with RSCUs > 1, most of which end with an A or T base. Therefore, we should give more attention to the codon usage preferences when carrying out molecular breeding based on CMS in the future.

### 3.2. RNA Editing

RNA editing events have potential effects on the development of plant CMS and can cause amino acid alterations in functional genes [31,37]. The exploration of RNA editing sites enhances our understanding of plant mitochondrial gene expression and provides insights for gene function prediction [22,52]. In our study, we predicted 360 RNA editing sites in the *V. bracteatum* mitogenome, with all of them taking the form of C-T conversions. Additionally, all predicted RNA editing sites occurred at the first and second codon positions [39]. Notably, RNA editing could change the start and stop PCG codons in the *V. bracteatum* mitogenome, thus altering gene function [34]. Typically, new start and stop codons resulting from RNA editing led to more evolutionarily conserved proteins, thus promoting mitochondrial gene expression [22,53]. Furthermore, the number of RNA editing sites differed greatly in different genes, with *ccmB*, *ccmC*, and *ccmFn* exhibiting higher frequencies of RNA editing events. However, RNA editing sites were predicted in almost all PCGs in the *V. bracteatum* mitogenome, thereby showing that RNA editing serves as a significant RNA-based regulatory layer and may play crucial roles in plant evolutionary adaptation and development [18]. In future work, we intend to identify RNA editing sites based on RNA-Seq data, and verify their accuracy.

### 3.3. Intergenomic Sequence Transfer Events

Another critical aspect of plant mitogenome evolution is widespread intracellular gene transfer or horizontal gene transfer [54]. Therefore, it is vital to track intergenomic transfer for comprehending the evolution of plant mitogenomes. With the development of genome research, gene transfer events among plant genomes (in the mitochondria, nuclei, and chloroplasts) have been uncovered [37]. Although the nu and cp genomes of *V. bracteatum* have been previously published, we obtained the complete mitogenome, which enabled us to make a comprehensive analysis for studying intergenomic sequence transfers of *V. bracteatum*.

In this study, extensive gene transfer between the nu and mt genomes was found in *V. bracteatum*. Every *V. bracteatum* chromosome experienced nuclear/mitochondrial transfer; however, the overall length and coverage differed among chromosomes. In regard to the migration from chloroplast genome to mitogenome, 5500 bp of transferred fragments were detected in *V. bracteatum*, comprising 0.78% of the mitogenome, which was significantly less than the previously reported data (1–12%) [27], but similar to *Punica granatum* (0.54%) [55], suggesting that sequence migration between cp and mt genomes varied greatly among different plants. Twenty-four chloroplast/mitochondrial fragments transferred, including six integrated genes, interestingly, four of which are tRNA genes. The migration of tRNA genes across different genomes is common in angiosperms, suggesting that tRNA genes are more conserved than PCGs and rRNA genes in the mitogenome, and they might still have normal transport functions and play an indispensable role in mitochondria [27,28,56]. Researchers have demonstrated that DNA transfer can lead to a high degree of rearrangements and enhance the diversity of the mitogenome [33,53]. However, the mechanisms driving sequence migration among the genomes remain unknown, and further research is required [22].

### 3.4. Mitogenome Comparison in Ericales Species

For a deeper insight into its structure and composition, the *V. bracteatum* mitogenome was compared to that of other Ericales species. The conservation analysis revealed notable variances in the types and number of PCGs within the species. Numerous gene losses in the mitogenomes of these Ericales species occurred commonly with ribosomal proteins and succinate dehydrogenase genes, but rarely occurred with respiratory genes [9,26,54]. However, the sets of core genes tend to be conservative, indicating that these genes may play a crucial role in plant function.

On the other hand, the majority of the PCGs in the *V. bracteatum* mitogenome experienced purifying selection throughout the evolutionary process, suggesting that the coding sequences were highly conserved, which was consistent with most of the angiosperm species reported [53,57,58]. Surprisingly, the genes *nad4L* and *atp1* were subjected to high conservation in Ericales plants, which may be essential for mitochondrial function. However, the mitogenome also had PCGs with Ka/Ks ratios > 1, such as *ccmB*, which might contribute significantly to future research on gene selection and evolution processes [42,52].

In addition, the plant mitogenome exhibited relative conservation in terms of the type, number, and sequence of functional genes; however, the arrangement of the mitogenome varied among different species. This variation may be attributed to extensive recombination events that occurred during the evolution of Ericales mitogenomes and promote the diversification and evolution of mitogenomes [57,58].

### 3.5. Phylogenetic Inference

Recent studies have characterized the genus *Vaccinium* based on genetic diversity and phylogenetic relationships [59,60,61], complete chloroplast genome assembly [62], genome size evolution [63], and nuclear genome assembly [24,64]. However, the taxonomy of the genus remains unclear, and the evolutionary relationship within this genus need to be further explored. To gain deeper insights into the evolution and divergence of plant lineages, it is crucial to thoroughly understand their genetic background, including their nuclear and organellar genomes [19]. Hence, assembling and analyzing the mitogenome could offer new insights for elucidating the phylogeny of *Vaccinium* [65]. 

In the current study, we conducted phylogenetic analyses within the Ericales order based on the available mitogenomes. Remarkably, our findings were highly consistent with the phylogeny of Ericales species in APG IV, which illustrated that mitogenomes could be used as signals for phylogenetic analyses. However, due to the lack of sufficient available mitogenomes, this is not enough to resolve the phylogeny and evolutionary biology within this large family. Therefore, more extensive mitogenomes of Ericaceae are needed to be sequenced for further research.

## 4. Materials and Methods

### 4.1. Raw Data Acquisition

The raw HiFi genome sequencing data employed for mitogenome and chloroplast genome assembly of *V. bracteatum* in this study were obtained from NCBI (SRR29004627), which were generated using SMRT sequencing on the PacBio Sequel II platform (Pacific Biosciences, CA, USA). An adult wild plant of the species *V. bracteatum* growing at Liyang County, Changzhou City, Jiangsu Province, China (119°23′27.66″ E, 31°24′41.10″ N) was used in the study.

### 4.2. Mitogenome Assembly and Validation

The assembly of the *V. bracteatum* mitogenome was performed as previously reported [46]. The software Minimap2 (v2.1) [66] was used to identify sequences >50 bp as candidate sequences in the alignment. Then, canu [67] v2.0 was employed for correcting the long-read sequencing data obtained. SPAdes v3.15.4 (https://github.com/ablab/spades; accessed on 3 January 2023) with the default parameter was used to stitch the corrected third-generation data, followed by using Bandage v0.8.1 (https://rrwick.github.io/Bandage/; accessed on 3 January 2023) to visualize and manually adjust the stitching results. Finally, minimap2 was employed to align corrected sequencing data to the count, and the final assembly results were obtained after manually checking.

To validate the genome assembly, we mapped the HiFi data with a length greater than 11 kb onto the complete mitogenome using minimap2, and the results were visualized using mummer4 [68] (Supplementary File S2: Figure S3). The entire assembly was covered with short reads at an average coverage of 135×, and the genome had no zero coverage, which could also certify that the genome was of high quality.

### 4.3. Mitogenome Annotation and Analysis

Mitogenome annotation and repeat sequences analysis followed previously reported method [46]. RNA editing sites in the PCGs of *V. bracteatum* were predicted using the PREP suite [69]. 

### 4.4. Homologous Fragment Analysis

To explore the potential homologous sequences among three genomes, we first utilized the same data for chloroplast genome assembly. The cp genome assembly and annotation method followed the approach described previously [70]. The annotated chloroplast genomes of *V. japonicum* (MW006668.1), *V. corymbosum* (OM791342.1), *V. virgatum* (OM791343.1), *V. myrtillus* (OM809159.1), and *V. floribundum* (OQ331035.1) were used as a reference. The annotated cp genome of *V. bracteatum* was deposited in GenBank (PQ284534).

The mitogenome was searched against the cp genome using BLASTN with matching rate  ≥  70%, E-value ≤ 1 × 10^−5^, and length  ≥  30 bp. To identify regions of potential nuclear origin in the mitogenome of *V. bracteatum*, we also performed a BLASTN search of the complete mitogenome against the nuclear genome of *V. bracteatum* sequenced in our previous study (SRR29004627) with matching rate ≥  80% and length  ≥  250 bp. Finally, data visualization was performed using circos v0.69-5 (http://circos.ca/software/download/; accessed on 3 January 2023).

### 4.5. Phylogenetic Tree Construction and Comparative Analysis

A total of 21 plant mitogenomes (Supplementary File S1: Table S13) were obtained from NCBI (http://www.ncbi.nlm.nih.gov/genome/organelle/; accessed on 4 January 2023). Utilizing MAFFT v7.427 [71], the 22 mitogenomes were compared and aligned. Following the end-to-end alignment, sequences were trimmed using trimAl v1.4.rev15, and jModelTest v2.1.10 (https://github.com/ddarriba/jmodeltest2; accessed on 4 January 2023) was employed to predict GTR status of the model. Finally, a maximum likelihood tree was generated with RAxML v8.2.10 (https://cme.h-its.org/exelixis/software.html; accessed on 4 January 2023). *Nicotiana tabacum* and *Lactuca serriola* were designated as an outgroup. The nucmer command (4.0.0beta2) in mummer4 was used for genome alignment and generating dot-plots. Genome synteny and rearrangements between *V. bracteatum* and *V. macrocarpon* mitogenomes were analyzed using in Mauve v2.4.0 [72]. The alignment of homologous gene pairs obtained using BLASTN was performed using MAFFT followed by calculation using Ka/Ks Calculator v2.0 [73]. MAFFT was used to compare homologous gene sequences. The Pi values among PCGs and rRNA genes were calculated by DnaSP v5 [74].

## 5. Conclusions

In this study, we assembled and annotated the complete mitogenome of *V. bracteatum* utilizing PacBio HiFi data for the first time. Furthermore, several mitogenome aspects have been studied, including nucleotide composition, codon usage, repeat sequences, migration sequences, and genome comparative analyses with closely related species. In addition, this report confirms that the mitogenome can provide new insights into phylogenetic evaluation. The complete decoding of the mitogenome completes the last link of the reference genome of *V. bracteatum*, which will be indispensable for evolutionary and genetic research, and establish a strong basis for molecular breeding, cultivation, and utilization.

## Figures and Tables

**Table 1:** Gene composition in the *V. bracteatum* mitogenome.

Group of Genes	Gene Name
ATP synthase	*atp1 atp4 atp6 atp8 atp9*
Cytohrome c biogenesis	ccmB ccmC ccmFc* ccmFn
Ubichinol cytochrome c reductase	*cob*
Cytochrome c oxidase	*cox1 cox2 cox3*
Maturase	*matR*
Transport membrane protein	*mttB*
NADH dehydrogenase	nad1**** nad2**** nad3 nad4** nad4L nad5**** nad6 nad7**** nad9
Ribosomal protein (LSU)	#rpl16 rpl10 rpl2* rpl5
Ribosomal protein (SSU)	#rps14 rps1* rps10*(2) rps12 rps13 rps19 rps3* rps4
Succinate dehydrogenase	*sdh4*
Ribosomal RNA	*rrn18 rrn26 rrn5*
Transfer RNA	trnC-GCA trnD-GTC trnE-TTC trnF-GAA trnG-GCC trnH-GTG trnK-TTT trnK-TTT* trnL-CAA(3) *trnM-CAT*(6) trnN-GTT trnP-TGG trnQ-TTG trnR-TCG*(2) *trnS-GCT trnS-TGA trnW-CCA trnY-GTA*

*: intron number; #Gene: Pseudo gene; the numbers in brackets are the number of copies.

**Table 2:** Mitogenome composition comparison of 7 Ericales species.

	Size (bp)	No of PCGs	No of rRNAs	No of tRNAs	No of Introns	AT%	GC%	AT Skew	GC Skew
*Diospyros oleifera*	493,958	39	3	27	29	54.30	45.70	0.00432	−0.00227
*Aegiceras corniculatum*	425,282	38	3	23	21	55.18	44.82	0.00163	0.00378
*Camellia sinensis*	707,441	32	2	23	14	54.25	45.75	−0.00214	−0.00175
*Actinidia latifolia*	825,163	40	3	20	24	53.84	46.16	−0.00186	−0.00094
*Rhododendron simsii*	802,707	41	4	23	22	54.13	45.87	0.00060	0.00405
*Vaccinium bracteatum*	708,384	36	3	26	27	54.72	45.28	−0.00203	0.00126
*Vaccinium macrocarpon*	459,678	33	3	18	18	54.67	45.33	0.00060	0.00246

## Data Availability

The annotated mitogenome of *Vaccinium bracteatum* has been deposited in the NCBI (https://www.ncbi.nlm.nih.gov/; accessed on 10 September 2024) with the accession number PQ283854.

## Supplementary Materials

The following supporting information can be downloaded at: https://www.mdpi.com/article/10.3390/ijms252212027/s1, Table S1: Composition and skewness of the *V. bracteatum* mitogenome; Table S2: Dispersed repeats in *V. bracteatum* mitogenome; Table S3: The SSR types in the *V. bracteatum* mitogenome; Table S4: Distribution of tandem repeats in *V. bracteatum* mitogenome; Table S5: Analysis of RNA editing sites and types in the mitogenome of *V. bracteatum*; Table S6: Composition and skewness of the *V. bracteatum* mitogenome; Table S7: RSCU values of PCGs in the *V. bracteatum* mitogenome; Table S8: Start codon and stop codon of PCGs of the *V. bracteatum* mitogenome; Table S9: Homologous fragments between the mt and nu genomes in *V. bracteatum*; Table S10: Homologous fragments between mt and cp genomes in *V. bracteatum*; Table S11: The Ka/Ks values of shared PCGs among 7 Ericales species; Table S12: The Pi values among Ericales mitogenomes; Table S13: The mitogenomes for the selected plants in this study; Figure S1: Distribution map of repetitive sequences in the *V. bracteatum* mitogenome. Figure S2: Gene order comparison between *V. bracteatum* and *V. macrocarpon*; Figure S3: The coverage of the HiFi data onto the *V. bracteatum* mitogenome.
