# Supplementary material for: Complete Mitogenome Assembly and Comparative Analysis of Vaccinium bracteatum (Ericaceae), a Rich Source of Health-Promoting Molecules
Source: Int J Mol Sci. 2024 Nov 8;25(22):12027. doi: 10.3390/ijms252212027 (PMC11593731; doi:10.3390/ijms252212027)
Supplement: Supplementary file 1 [file ijms-25-12027-s001.zip › Supplementary Figures.pdf]

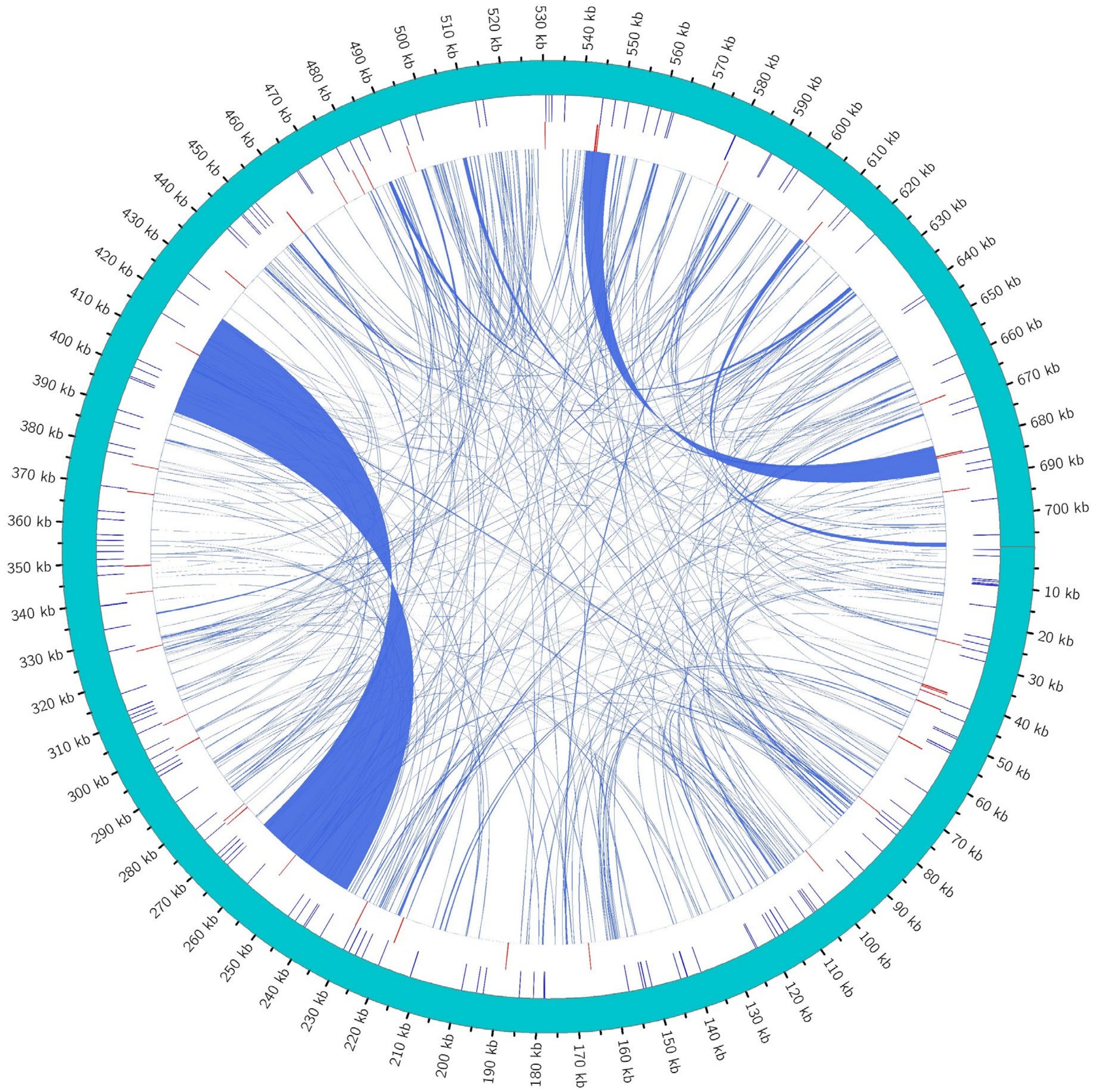

Figure S1. Distribution map of repetitive sequences in the *V. bracteatum* mitogenome. The outermost circle is the SSRs, followed by the tandem repeat sequence, and the innermost concatenation is the dispersed repeat sequence.

Figure S2. Gene order comparison between *V. bracteatum* and *V. macrocarpon*.

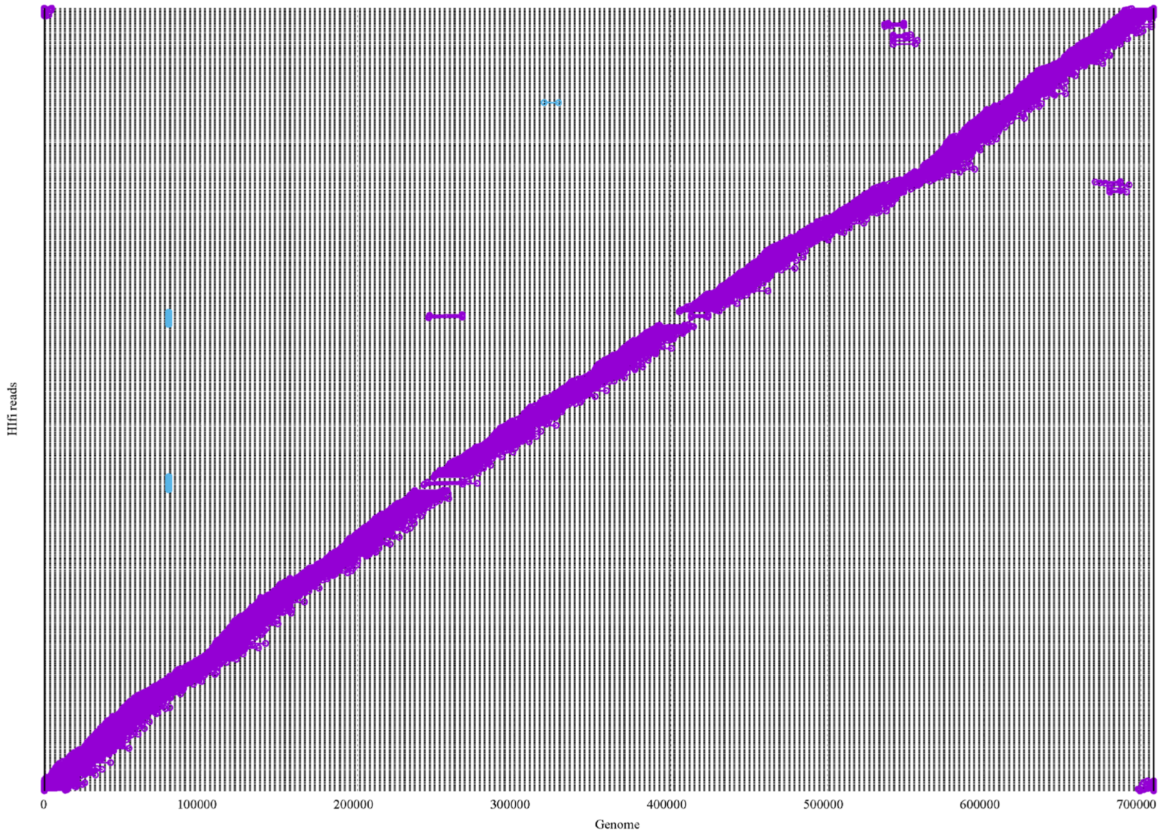

Figure S3. The coverage of the HiFi data onto the *V. bracteatum* mitogenome.
